# Prevention of data duplication for high throughput sequencing repositories

**DOI:** 10.1093/database/bay008

**Published:** 2018-02-27

**Authors:** Idan Gabdank, Esther T Chan, Jean M Davidson, Jason A Hilton, Carrie A Davis, Ulugbek K Baymuradov, Aditi Narayanan, Kathrina C Onate, Keenan Graham, Stuart R Miyasato, Timothy R Dreszer, J Seth Strattan, Otto Jolanki, Forrest Y Tanaka, Benjamin C Hitz, Cricket A Sloan, J Michael Cherry

**Affiliations:** Department of Genetics, Stanford University, Stanford, CA 94305-5120, USA

## Abstract

Prevention of unintended duplication is one of the ongoing challenges many databases have to address. Working with high-throughput sequencing data, the complexity of that challenge increases with the complexity of the definition of a duplicate. In a computational data model, a data object represents a real entity like a reagent or a biosample. This representation is similar to how a card represents a book in a paper library catalog. Duplicated data objects not only waste storage, they can mislead users into assuming the model represents more than the single entity. Even if it is clear that two objects represent a single entity, data duplication opens the door to potential inconsistencies between the objects since the content of the duplicated objects can be updated independently, allowing divergence of the metadata associated with the objects. Analogously to a situation in which a catalog in a paper library would contain by mistake two cards for a single copy of a book. If these cards are listing simultaneously two different individuals as current book borrowers, it would be difficult to determine which borrower (out of the two listed) actually has the book. Unfortunately, in a large database with multiple submitters, unintended duplication is to be expected. In this article, we present three principal guidelines the Encyclopedia of DNA Elements (ENCODE) Portal follows in order to prevent unintended duplication of both actual files and data objects: definition of identifiable data objects (I), object uniqueness validation (II) and de-duplication mechanism (III). In addition to explaining our modus operandi, we elaborate on the methods used for identification of sequencing data files. Comparison of the approach taken by the ENCODE Portal vs other widely used biological data repositories is provided.

**Database URL**: https://www.encodeproject.org/

## Introduction

Authors of scientific publications are commonly required by journals to deposit their published data in public repositories [i.e. GEO and SRA at the NCBI (1)], allowing access for the wider scientific community and ensuring data preservation. These repositories have to handle an exponentially growing amount of sequencing data (https://www.nature.com/scitable/topicpage/genomic-data-resources-challenges-and-promises-743721) produced by different research laboratories.

Information submitted to these repositories is subjected to data validation processes that include syntactic validation of the submitted components, file integrity checks and manual curation [e.g. see GEO (2) or GDC https://gdc.cancer.gov/submit-data/gdc-data-submission-portal submission practices]. The large volume of submitted data makes it impossible to extensively curate each submitted data set manually (https://www.nature.com/scitable/topicpage/genomic-data-resources-challenges-and-promises-743721) and while file formats can be readily validated computationally in an automated fashion, evaluation of the actual file content is considerably more difficult and computationally expensive.

The Encyclopedia of DNA Elements (ENCODE) Portal (https://www.encodeproject.org) is a database that is funded by the NIH to organize, curate and share ENCODE data (3). It also hosts related projects like Genomics of Gene Regulation (GGRhttps://www.genome.gov/27561317/genomics-of-gene-regulation/) and modERN (modERN Project: https://grants.nih.gov/grants/guide/pa-files/PAR-11-095.html), as well as the imported REMC (4) and modENCODE (5) data. As of October 2017, the ENCODE Portal houses data from >50 assays and >600 different cell and tissue types. The data and metadata are submitted to the ENCODE Data Coordination Center (DCC) whose role is to validate, describe, organize and provide access to these diverse data sets (6). High quality and easily accessible data, tools and analyses are provided to the scientific community via the ENCODE Portal. Additionally, these data are deposited to external repositories (e.g. GEO and SRA repositories, which belong to the list of official NIH Data Repositories and Trusted Partners https://osp.od.nih.gov/scientific-sharing/data-repositories-and-trusted-partners/).

The process of collection, representation and distribution of an extensive and exponentially growing amount of high-throughput genomic data is challenging. To face these challenges, the ENCODE DCC has developed metadata organization principles and standards that were described by Hong *et al.* (3). One of the challenges scientific data repositories in general and ENCODE DCC in particular face is unintended data duplication. The duplication can occur in the data itself or in the object’s metadata, akin to a presence of multiple copies of the same book in the library in the former case and presence of multiple cards in the library catalog in the latter. The distinction between data (file content) duplication and metadata (objects) duplication is significant for many reasons. For Next-generation Sequencing (NGS), the data files can reach the size of 10 s to 100 s of gigabytes, therefore, the wasted storage capacity from the duplication of data can be substantial, approaching thousands of US dollars per annum for a large project (based on approximate cloud storage price of $22.50/TB/month). Duplication of data can have the more scientifically relevant consequence of confounding downstream integrative analyses. If for example, the same sequencing run is submitted multiple times, incorrect assumptions might be made about the overall read depth of the experiment. Similarly, and perhaps even more importantly, analysis of experimental replicates associated with duplicated data files would result in erroneous and misleading high correlation scores between the replicates. If in the library analogy, the downstream analysis was based on words per title, duplicate copies of a book would confound the analysis. Additionally, prevention of data duplication is another quality check on the data. Just as the same set of sentences showing up in two different books is a sign of potential plagiarism, when the same set of sequencing reads appears in a heart experiment and in a liver experiment, there has been an error in submission. Fortunately, much thought has been put into prevention of data duplication (e.g. the Groveler process from https://www.microsoft.com/en-us/research/wp-content/uploads/2000/01/WSS2000.pdf) and those methods can be built upon for the specifics of sequencing data. Unlike for data duplication, the wasted storage from metadata duplication can be insignificant. However, metadata duplication can have a profound impact on interpretation and integrative analysis. For example, the conclusions of analyses performed on samples taken from two different donors would differ from the conclusions one would reach if the samples were coming from the same donor. Additionally, the duplication of records creates a maintenance burden by creating a situation in which any update to the data record is an opportunity to create inconsistencies between duplicated records. The larger the number of inconsistencies between the duplicated and initially identical records, the harder it is to automatically consolidate. Finally, whereas there are established methods for determining data uniqueness, metadata uniqueness rules are individual to the data type and model. This makes duplication prevention in metadata more elusive.

Here, we describe in detail ENCODE DCC metadata organization guidelines and validation approaches preventing unintended data duplication.

## Guidelines

### Identifiable data objects definition

A well-defined object in the data model should reflect an actual experimental/physical entity, rather than an abstract concept. The object should contain properties that could be used in maintenance of a one-to-one correspondence between an object and the entity it represents. Abstract concept elements often lack such properties that would allow their uniqueness to be checked. For example, a definition of an object representing a library book in a data model allows check for uniqueness, because we can collect book identifying properties (such as a library catalog number) and require uniqueness of these properties in the system. But, a definition of an object representing a classification system (like Dewey Decimal) would be difficult to check for uniqueness since the only identifying characteristic is its name.

### Object uniqueness validation

Prevention of duplication and preservation of one-to-one correspondence between the data objects and the entities represented by these objects can be achieved by defining a set of properties that make the data object unique and enforcing that each object be unique for those properties. Using our library analogy, a set of properties that would allow uniqueness validation for a book would include author name, book title, publisher name, publication date and book copy number. The unique combination of these properties defines book object uniqueness in the system.

### De-duplication mechanism

For cases in which violation of one-to-one correspondence between the data object and the represented entity is detected, the system should have a mechanism in place that would allow resolution of the duplication event. In full agreement with McMurry *et al.* (7), we think that publicly exposed identifiers should not be deleted or reassigned. Therefore, duplication events should be resolved using merging and redirection mechanisms that preserve both existing identifiers. Situations in which two library catalog cards are found to be for the same book should be resolved by leaving only one functional card, marking the rest of the cards as deprecated and forwarding to the single functional card.

## Implementation according to encode DCC guidelines

### Definition of identifiable data objects

**Figure 1.**
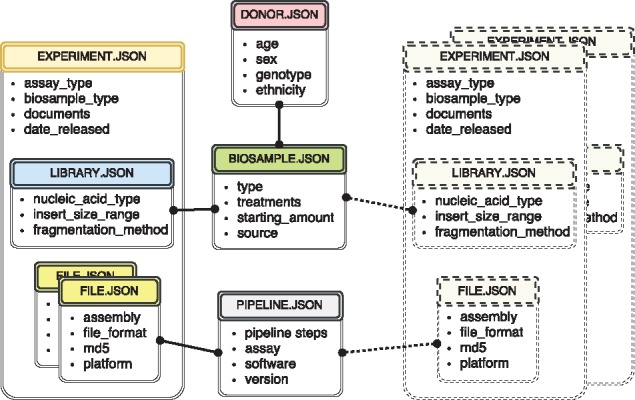
The metadata captured for ENCODE can be grouped into the following main object types: donors/strains, biosamples, genetic modifications, sequencing libraries, antibodies, data files and pipelines. Experiment objects (representing replicates of an assay) are constructed from these object types. Each object type represents a category of experimental entities and is used to store information about entities from that category. For example, the library object represents sequencing library and would include information such as nucleic acid type or the fragmentation method used to construct the library. In a similar fashion the file object (that is different from the actual data file) stores information about the data file submitted to the portal. Examples of the properties that would be stored in a file object would be: information about the sequencing platform used to produce this FASTQ file or information about the reference genome assembly that was used for alignment producing this BAM file. Some of the objects are unique per experiment (e.g. sequencing library, or raw data file) while others could be shared between different experiment objects (e.g. the donor or the biosample objects). The figure includes both types of objects, the library (blue color header) and the files (yellow color header) are unique and are associated with a single experiment (amber header), while the biosample (green color header) and the pipeline (pink color header) objects could be shared between multiple experiments. Potential experiments the biosample and the pipeline objects could be shared with are depicted by the rectangles with the dashed borderline. Only a subset of object types is listed in the figure to provide an overview of the breadth and depth of metadata collected. The full set of metadata can be viewed at https://github.com/ENCODE-DCC/encoded/tree/master/src/encoded/schemas.

The metadata used at the ENCODE portal is organized in a set of major categories that expands the properties collected during previous phases of the ENCODE project (3, 8). The current set includes donors, biosamples, genetic modifications, DNA libraries, antibodies, experiments, data files and analysis pipelines. For more detailed description of the data model objects, see (3) and https://www.encodeproject.org/help/getting-started/. Wherever possible, the objects correspond to simply defined ‘real-world’ entities. For example, an antibody lot can be defined as corresponding to a specific monoclonal antibody clone. A donor is an individual human. A biosample is a particular harvest of a cell line or a tissue. A library specifies the DNA library generated from an individual assay. Just like the book in the traditional library, there should be a tube in the lab that corresponds to the created record. For more conceptual entities, like experiment and analysis pipeline, the submitters need as much educational material as possible on the definition of that object. Ideally, there is a collection of properties that can uniquely identify that object. For example, cell-type, harvest date, donor and source could define a biosample. If two biosamples are harvested together, introducing collection time would allow unique identification. Our approach for data modeling is exemplified by a subset of metadata objects included in Figure 1.

### Object uniqueness validation

Creation of each new object in our site generates a new unique identifier. However, generation of a new unique identifier by itself cannot prevent an element’s duplication, as it is possible to violate the one-to-one correspondence by the creation of multiple objects with different identifiers that correspond to a single entity. For this reason, we are always looking to add uniquely identifying properties to an object definition.

Whenever possible, the ENCODE DCC leverages existing unique and uniformly assigned identifiers for an entity. For example, if a cell line is defined in Cellosoaurus (http://web.expasy.org/cellosaurus/) or a mouse strain is defined by The Jackson Laboratory (https://www.jax.org/), the schemas accept those identifiers as uniquely identifying aliases in our system. Even if the identifier is only unique to the submitting lab, our schema allows for the addition of these unique identifiers. When creating a new object in our system, the DCC encourages submission of as many of these identifiers as possible, allowing both for uniqueness check based on the uniqueness of external identifier and interoperability between different data resources. As it is noted in (7), use of external identifiers allows for preservation of a one-to-one correspondence between an identifier and an entity, preventing future costly mapping problems. Additionally, we recommend where possible using a combination of multiple properties that would result in a unique object. For instance, one could use biosample, producer, assay and date to define a particular library.

**Figure 2.**
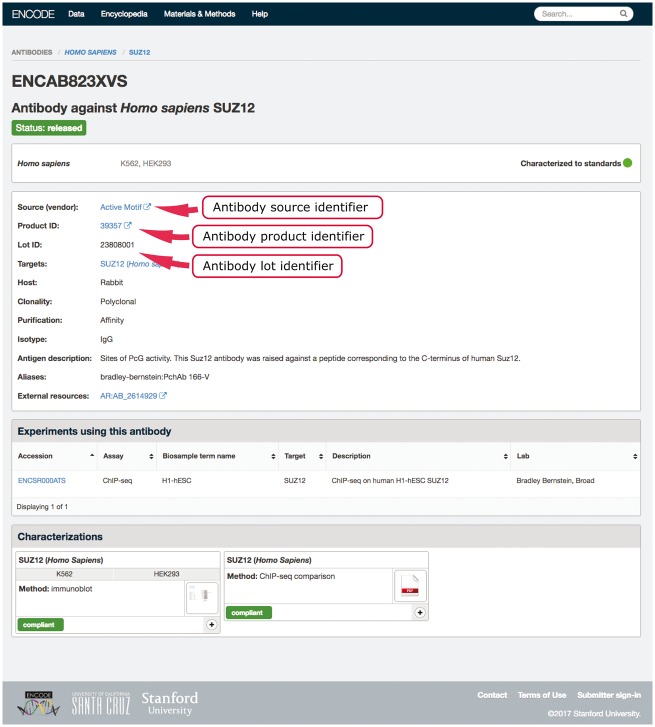
Example of an antibody page for antibody lot (ENCAB823XVS). Various aspects of metadata are displayed on the antibody page, including the properties that are used for antibody uniqueness validation. The set includes the lot identifier, product identifier and the source (vendor) name.

An example in our system of using multiple properties to ensure uniqueness is our antibody-lot object. For this object, we require that the combination of source, product ID and lot number be unique (Figure 2). If a submitter tries to create a new object with the same values for those three properties as an existing object, they will get an error message regarding the duplication. This allows them to reference the existing antibody-lot object as referring to their actual entity. However, the definition of lot varies from company to company. In some cases, it was discovered that the same antibody clone was being sold with differing product numbers based on the volume of the purchased tube. For this reason, the ability to merge those two entries later is essential. As a further attempt to clearly and uniquely identify antibody lots, we work with antibodypedia (9) to provide external identifiers for cross-referencing.

**Figure 3.**
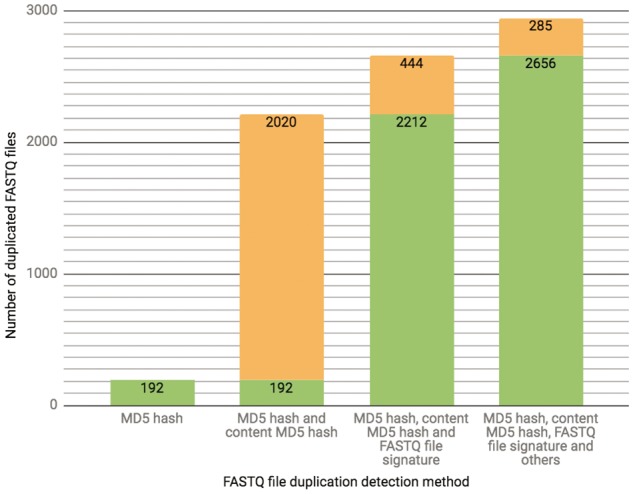
The chart presents the breakdown of the total number (2941 as of 10/13/2017) of FASTQ file duplication events detected in our database using different methods. Each successive bar shows the split between the number of files that could be detected with the methods from the previous bar (depicted in a green color) as well as those that could only be detected with the additional method (depicted in an orange color). The final 285 FASTQ files were considered duplicates based on manual curation.

The ENCODE Portal is the only repository for the majority of ENCODE-generated files, and thus these files lack an external identifier, such as a SRR identifier that would come from an initial submission of primary data to SRA. In addition to a unique accession the file objects receive on the portal [for details see (3)], we calculate file metadata values in an effort to mitigate cases of unintended file duplication, which are highly probable when the repository hosts >380 000 files. For example, the current set of properties calculated for verification of a FASTQ file uniqueness is a result of an ongoing iterative trial and error process. Initially, we used a MD5 hash function (10) as a way to generate a compact digital fingerprint of a file for fast and reliable file comparisons. However, calculation of MD5 hash function of compressed (gzipped) files turned out to be unreliable source for the file comparison due to the introduction of the timestamp in the header of the compressed version of the file by gzip compression algorithm. Unless default settings are explicitly over-ridden, a file compressed (gzipped) at different time points will have different timestamps in the header, leading to different MD5 hash function results, obscuring the fact that the original content was identical. To overcome this difficulty, we first un-compress FASTQ files and calculate the MD5 hash function using the uncompressed file, forcing creation of a digital file fingerprint reflecting the actual file content rather than the compressed file content (for numerical details see Figure 3).

Unfortunately, although comparison of content MD5 hash function results ensures the uniqueness of the digital file, it still does not ensure uniqueness of the content. Specifically, two FASTQ files could contain identical reads but in a different sort order. In this case, the file’s MD5 hash function results would be different, but the content (defined as a unique collection of sequencing reads) would be the same and should be considered a duplication of a sequencing event. Additionally, the reads could be the same and in the same order, but the read names could have some minor modification made by the submitting lab. The lack of an enforced standard sequencing read naming convention paired with the diversity of technologies used to produce those reads leaves a substantial chance for variation in read names.

**Figure 4.**
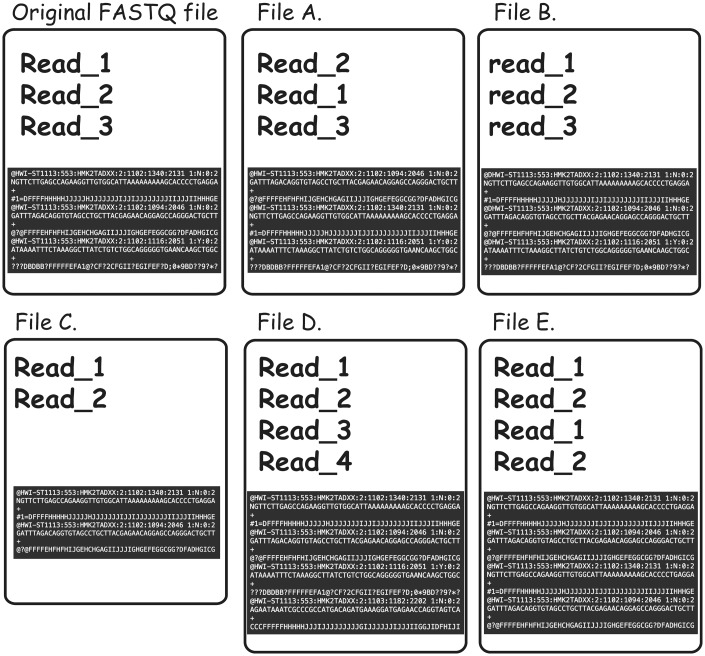
Types of FASTQ file duplications detectable using FASTQ signature heuristic. It is important to note that all the cases presented here are not detectable using MD5 hash or content MD5 hash function approaches, as those functions’ results will be different from the original FASTQ file for all listed files. **File A** represents a case where reads from original file are out of order. FASTQ signature heuristic would detect duplication of this type. **File B** is identical to the original file, except for a small change in read names making detection of file content duplication challenging. Since FASTQ signatures are constructed using only parts of the read name, the ability of the heuristic to detect duplication will rely on the exact places the read names were modified. **File C** contains subset of the reads from original file and will be detected by FASTQ signature heuristic. **File D** contains reads not present in original file; however, it will be reported as a potential duplication because it contains reads identical to the content of original FASTQ file. **File E** will be reported as duplication of the original FASTQ file, since Read_1 and Read_2 appear in both files. However, File E contains both internal duplication and external duplication of the original FASTQ file. The internal duplication is not detectable using our current FASTQ signature approach.

To further complicate the issue, non-identical FASTQ files may contain partial duplication, which would not be detectable even in cases in which both of the files in question contain sequencing reads in the same order and the read names were not altered. For instance, content MD5 hash function would not be useful in cases when some of the reads were omitted from one of the files for reasons of filtration or truncation (examples of the different types of FASTQ duplication detectable using FASTQ signature heuristic are presented in Figure 4).

Theoretically, read uniqueness could be checked on an all-by-all basis, but this is not practical due to the large quantity of reads in each file (millions of reads) and the quantity of FASTQ files stored in the system (>51 000). In order to make the comparison feasible, we use a heuristic allowing us to produce a value we call ‘FASTQ signature’ that can be compared against other FASTQ files’ signatures in a timely fashion and in most cases, would ensure file uniqueness.

### Description of the FASTQ signature calculation heuristic

According to the specification of the FASTQ format (https://support.illumina.com/content/dam/illumina-support/documents/documentation/software_documentation/bcl2fastq/bcl2fastq_letterbooklet_15038058brpmi.pdf), each sequencing read in the FASTQ file has an identifier, called also a read name. Starting from Illumina CASAVA 1.8 software identifier in FASTQ file will appear in the following format:

@<instrument>:<run number>:<flowcell ID>:<lane>:<tile>:<xpos>:<y-pos> <read>:<is filtered>:<control number>:<barcode sequence>

**Figure 5.**
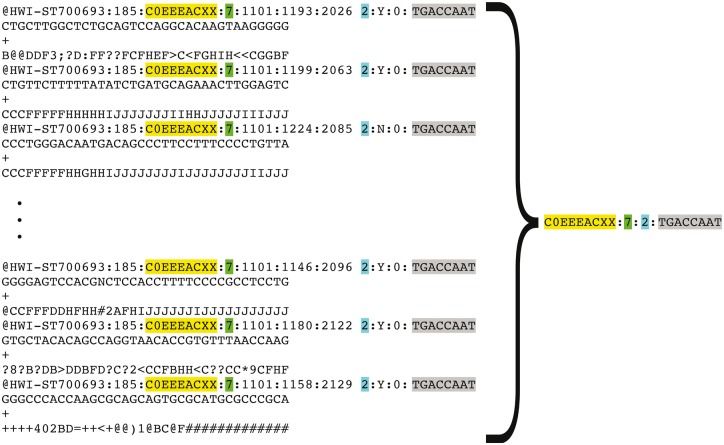
Representation of FASTQ file content by FASTQ signature. FASTQ signature is constructed using read name parts that are common for multiple reads within a single FASTQ file. Read name parts that are used for signature construction are color coded in the figure: flowcell identifier (yellow), flowcell lane number (green), read 1 or read 2 (turquoise) and index sequence (grey). Our condensation approach allows representation of multiple reads in FASTQ file by a single FASTQ signature, as it is exemplified in the figure.

Some of the read name parts listed in Table 1 would be unique for every sequencing read (like the combination of the tile and cluster coordinates), while other parts would be common to multiple reads in the FASTQ file created for a sequencing run on a specific Illumina instrument (like the machine name, flow cell id, lane number). For every FASTQ file, we retain sets of read name properties that are common for multiple reads in the file, but are unique for the file in question. This process allows us to represent a file containing millions of sequencing reads by a short list of unique strings constructed from the retained sets of read name parts (see Figure 5).

**Table 1.** Read name (sequence identifier) elements

Element	Requirement	Description
@	@	Each sequence identifier line starts with @
<instrument>	Characters allowed: a–z, A–Z, 0–9 and underscore	Instrument ID
<run number>	Numerical	Run number on instrument
<flowcell ID>	Characters allowed: a–z, A–Z, 0–9	Flowcell identifier
<lane>	Numerical	Lane number
<tile>	Numerical	Tile number
<x_pos>	Numerical	X coordinate of cluster
<y_pos>	Numerical	Y coordinate of cluster
<read>	Numerical	Read number. 1 can be single read or read 2 of paired-end
<is filtered>	Y or N	Y if the read is filtered did not pass), N otherwise
<control number>	Numerical	0 when none of the control bits are on, otherwise it is an even number
<barcode sequence>	ACTGCA	Barcode sequence

The table is from Illumina ‘bcl2fastq User Guide’ documentation https://support.illumina.com/content/dam/illumina-support/documents/documentation/software_documentation/bcl2fastq/bcl2fastq_letterbooklet_15038058brpmi.pdf.

Representation of a FASTQ file containing millions of reads by a short list of unique FASTQ signatures makes comparing a new file to all existing files in the repository feasible, due to the substantial reduction in the number of comparisons needed to detect file content duplication (full or partial).

During the application of our approach we encountered some difficulties:

- A fraction of the files on the ENCODE Portal were produced by software that is the predecessor of Illumina CASAVA 1.8 software and have read names that do not follow the format that could be used to produce FASTQ signatures.
- As mentioned before, because the read names format is not enforced, some of the files have their read names altered by the submitters prior to submission to the DCC.
- The introduction of libraries constructed using Unique Molecular Identifiers (UMI) (11) doesn’t allow us to use the index property as a common property shared between many read names in a given file.

To overcome these difficulties, we are constantly upgrading and updating the FASTQ signature calculation algorithm and encouraging production laboratories to submit raw FASTQ files containing unaltered read names, which are produced by the CASAVA software and have read names constructed according to the FASTQ format specifications by default. The code that is used for FASTQ signature calculation is freely accessible at https://github.com/ENCODE-DCC/checkfiles.

### De-duplication mechanism

**Figure 6.**
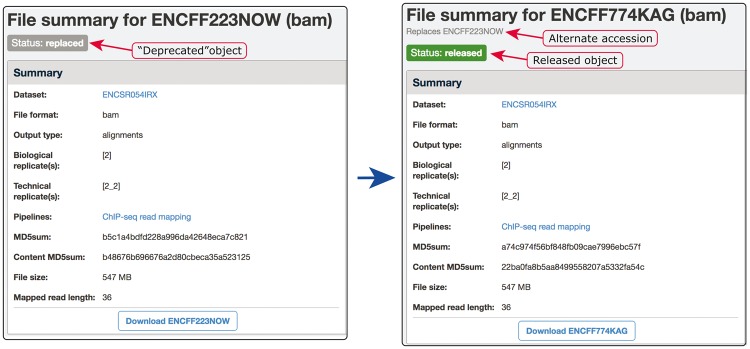
Example of our de-duplication mechanism. BAM files ENCFF223NOW and ENCFF774KAG were found to be data objects representing the same file. To resolve this situation of file objects duplication, ENCFF223NOW was deprecated (status changed to ‘replaced’) and the accession ENCFF223NOW was added to the list of alternate accessions of the file ENCFF774KAG. Searches for ENCFF223NOW are automatically redirected to the file ENCFF74KAG.

Although multiple objects representing a single entity are problematic for the reasons stated above, we believe that ‘released’ (publicly available) accessions should not be deleted or reassigned to new objects. The logic is similar to that of Lesson 7 from (7): publicly exposed identifiers may be deprecated, but must never be deleted or reassigned to another object. However, if two identifiers are referring to the same real-world entity, there needs to be a mechanism to ‘merge’ the data objects associated with those identifiers. Our system has a mechanism in place to resolve situations where one-to-one correspondence between an object and its represented entity is violated. For example, if we discover two donor objects representing the same human donor, we would want to resolve this situation and ultimately to have one object representing one human donor. In these cases, the objects are ‘merged’. All but one of the duplicated objects are switched into status ‘replaced’ and their accessions are added as ‘alternate’ accessions to the single object that remains. These ‘replaced’ objects are no longer available to the public and can only be seen by data curators. For all public users, accessions of the ‘replaced’ objects resolve via redirection to the remaining object. Effectively, all the accessions remain as identifiers to the one remaining object. In this way, old accessions are still valid and seamlessly redirect the user to the current ‘unique’ data object. For a detailed example see Figure 6. Different categories of duplication events on the ENCODE portal (Table 2 presents an enumeration of the duplication events, grouped by object type) are analysed, leading to development of validation algorithms that could be used to prevent the duplication from entering the system.

**Table 2.** Replaced objects on the ENCODE Portal

Object type	Number of objects with status ‘replaced’ as of 10/13/2017	Total number of objects
Donor	166 (8.3%)	2001
Biosample	327 (2.2%)	15 192
Library	193 (0.7%)	29 021
Antibody	43 (1.3%)	3373
File (FASTQ)	2941 (5.6%)	52 421
File (non-FASTQ)	7085 (1.9%)	365 203
Data set	488 (1.9%)	25 641
Pipeline	3 (5.5%)	54

This table enumerates the duplication events detected on the ENCODE Portal, grouped by object type as of 10/13/2017. As we discover patterns of duplication we attempt to prevent it from ever entering the system.

## Comparison with other data repositories

The desire to allow wide experimental diversity to be represented in a single repository along with the pressure from the scientific community to make the data submission process as fast and as easy as possible have taken their toll on the level of validation and scrutiny data submission undergoes. Relaxed data integrity checks can cause data duplication and other types of submission errors, correction of which would require substantial curation effort.

NIH NCI GDC (12) data model components are defined by GDC Data Dictionary (https://docs.gdc.cancer.gov/Data_Dictionary/). All data submissions are validated by GDC API against the Data Dictionary, detecting potential errors and invalid entities, which are not further processed until they are corrected and re-uploaded by the submitters. There is no automatic file uniqueness validation method in place. NCBI’s GEO’s (1) submission process (https://www.ncbi.nlm.nih.gov/books/NBK159736) is described as syntactic validation followed by manual curation review. Files belonging to the same experimental series and submitted under the same GSE identifier are checked for uniqueness using MD5 hash function or checksum (UNIX cksum command) calculation, but they are intentionally not checked against the whole database to allow for the duplicate submission of controls. The BAM files submitted to SRA are checked for uniqueness using the combination of a filename and MD5 hash function calculation result. Although the utilization of an MD5 hash function is a step in the right direction, we find that the majority of file content duplication events for FASTQ files, the *de facto* standard file format type for high throughput sequencing experiments, cannot be detected using this method (see Figure 3).

In cases where multiple distinct records (unique identifiers) are representing a single entity, there is a need for a mechanism for ambiguity resolution. The HUGO Gene Nomenclature Committee (http://www.genenames.org/) handles situations where more than one HGNC ID is associated with single genomic locus by turning one of the records to status ‘symbol withdrawn’ and redirecting any search for the withdrawn symbol to the ID of the symbol that remains in status ‘approved’, for example, AGAP8 was merged into AGAP4 (http://www.genenames.org/cgi-bin/gene_symbol_report? hgnc_id=23464). NIH NCI GDC describes handling of erroneous data in the following way: ‘Erroneous Data – If any available GDC data is discovered to be incorrect, the GDC will in general work with the submitter to revise and release a corrected version. In unusual situations, in particular if it is discovered that genomic data is incorrectly mapped to case or biospecimen data in a way that cannot be resolved by remapping, all affected data may be made indefinitely unavailable. The GDC will attempt to work with the submitter to resolve such issues without removing data if possible.’

Erroneous data detected in GEO in most cases would not be deleted. Instead, a comment indicating the problem would be added to the record, as stated on the GEO website https://www.ncbi.nlm.nih.gov/geo/info/update.html: ‘Please keep in mind that updating records is preferable to deleting records. If the accessions in question have been published in a manuscript, we cannot delete the records. Rather, a comment will be added to the record indicating the reason the submitter requested withdrawal of the data, and the record content adjusted/deleted accordingly.’

The three examples above demonstrate the diversity of ways different databases handle duplication events among other types of data errors. Until the scientific community as a whole adopt and enforce the best practices and common standards similar to what is described in (7), different repositories will continue to handle these situation in non-uniform fashion.

## Discussion

Since the amount of the scientific data submitted to genomic research databases is growing exponentially (https://www.nature.com/scitable/topicpage/genomic-data-resources-challenges-and-promises-743721), the fraction of the data that can be subjected to manual curation will only decrease with time. In order to ensure high quality of the data being submitted to the scientific data repositories, the submission process has to be automated and depend less on human intervention. It can be done without sacrificing data quality only if the scientific community as a whole (including researchers, funding agencies, journal publishers and data repositories) develops best practices for data handling and submission and adopts uniform data standards that can be enforced during the data submission process, without dependence on a specific repository. This would not only allow automated curation, but would also make submitters’ work easier, because instead of the need to comply with a repository-specific set of requirements, all of the repositories would have the same requirements. FAIR data principles (13), best practices for large-scale data integration (7) and the duplication prevention approaches described in this article are all steps in that direction and hopefully will encourage others to join the effort to ensure the efficient storage and re-usability of high quality data in various repositories.

## Funding

National Human Genome Research Institute at the National Institutes of Health [grant numbers U24 HG009397, U41 HG006992]. The content is solely the responsibility of the authors and does not necessarily represent the official views of the National Human Genome Research Institute or the National Institutes of Health. The funders had no role in design, data processing, implementation, decision to publish or preparation of the manuscript. Funding for open access charge: U24 grant from the National Human Genome Research Institute at the United States National Institutes of Health [U24 HG009397].
